# A Rare Case of Drug-Resistant *Nocardia transvalensis* Infection in a Renal Transplant Patient

**DOI:** 10.1177/2324709620909243

**Published:** 2020-02-28

**Authors:** Rajan Kapoor, Sreedhar Adapa, Anusha Vakiti, Imran Yaseen Gani, Laura Mulloy, Sandeep Anand Padala

**Affiliations:** 1 Augusta University Medical Center, Augusta, GA, USA; 2 Kaweah Delta Medical Center, Visalia, CA, USA

**Keywords:** kidney transplant, *Nocardia*, *Nocardia transvalensis*, disseminated nocardiosis, multidrug-resistant *Nocardia transvalensis*, pulmonary nocardiosis, immunocompromised, linezolid

## Abstract

*Nocardia transvalensis* is a rare species of *Nocardia* and is known to be a drug-resistant organism. Multiple cases have been reported of *Nocardia* species causing opportunistic infections in immunocompromised hosts. To our knowledge, we report the first case of successfully treated drug-resistant *Nocardia transvalensis* causing pulmonary nocardiosis in a renal transplant patient. Our case validates the importance of prompt identification of *Nocardia* species and their drug sensitivities to improve clinical outcomes and reduce mortality.

## Case Presentation

A 69-year-old male with past medical history of hypertension, asthma, coronary artery disease, end-stage renal disease secondary to hypertensive glomerulosclerosis s/p deceased donor kidney transplant 4 months back presented to the hospital with productive cough, fever, and anorexia of 2 weeks duration. Vitals on presentation were temperature of 38.5°C, heart rate of 72/min, blood pressure of 167/62 mm Hg, and respiratory rate of 22/min with oxygen saturation between 90% and 92% on room air. On physical examination, he had bibasilar crackles, more prominent on the left. Laboratory findings revealed an elevated white blood cell count of 15 100/µL, blood urea nitrogen of 21, and creatinine of 1.9 (baseline creatinine of 1.9-2.0). Urinalysis was unremarkable. Chest X-ray (CXR) showed a left lower lobe consolidation. Pan cultures were obtained, and treatment was started with intravenous vancomycin and piperacillin/tazobactam for empiric coverage. His posttransplant immunosuppressive regimen of mycophenolate mofetil, tacrolimus, and prednisone was continued along with other supportive measures. His prophylactic medications of nystatin, valganciclovir, and trimethoprim-sulfamethoxazole (TMP-SMX) were discontinued 4 weeks prior to admission after completion of 3-month posttransplant prophylaxis course. On day 3, 1 out of the 4 blood cultures grew gram-positive bacilli for which ampicillin was added for better central nervous system (CNS) penetration to cover *Listeria* infection as well. The computed tomography (CT) scan of head and lumbar puncture ruled out any CNS involvement. On day 7, final report of the blood culture revealed weakly acid-fast filamentous gram-positive bacteria. On further review of the gram stain and colony morphology of the organisms, it was identified as *Nocardia*. The diagnosis of disseminated nocardiosis secondary to pulmonary source (left lower lobe pneumonia) was made. Treatment for *Nocardia* was initiated with imipenem/cilastatin, and initial antibiotics (vancomycin, piperacillin/tazobactam, and ampicillin) were discontinued. Despite changing the antibiotics, there was no significant improvement in his overall clinical condition, and the patient remained with supplemental oxygen–dependent, persistent cough and with productive phlegm. Speciation of the organism was consistent with *Nocardia transvalensis*, which was resistant to imipenem, bactrim, ceftriaxone, augmentin, and minocycline. It was reported to be sensitive to linezolid, amikacin, moxifloxacin, clarithromycin, and tobramycin. Imipenem/cilastatin was discontinued, and linezolid along with moxifloxacin was initiated on day 14. Within the next 3 days, the patient started showing signs of improvement and was discharged home in a stable condition. He took linezolid 300 mg daily and moxifloxacin 400 mg daily for 12 months and remained asymptomatic. His follow-up visits were uneventful, and the patient was asymptomatic. Repeat CXR at the follow-up visit showed resolution of the pneumonia.

## Discussion

Renal transplant patients are at a higher risk of infections as compared with the general population due to higher degree of immunosuppression posttransplant. Bacterial, viral, and fungal infections are all frequently seen in patients in the posttransplantation period. Bacterial infections causing urinary tract infections, diarrhea, pneumonia, and wound infections are the most common scenarios^1^ in the early posttransplant period. Highly invasive fungal infections have also been reported within first 4 weeks of transplantation,^2^ and concurrent infections with fungal and viral etiology have also been reported within first 12 months of transplant.^3^ *Nocardia* is a ubiquitous, slow-growing, variably acid-fast, gram-positive *Bacillus*. It appears as a filamentous bacterium with branching hyphae and is commonly found in soil, fresh as well as saltwater, and decomposing vegetation. *Nocardia* usually manifests as an opportunistic infection in immunocompromised hosts.^4^ Risk factors for *Nocardia* infection include impaired cell-mediated immunity states such as solid organ transplant or hematopoietic stem cell transplant recipients, lymphoma and other malignancies, human immunodeficiency virus (HIV) infection, cytomegalovirus infections, and patients with chronic obstructive pulmonary disease and diabetes.^5,6^ As per a review of 303 cases of nocardioses from Japan, the most common predisposing factors were immunosuppressive agents (22.4%), cancer (6.6%), diabetes (3.6%), and AIDS (2.0%).^7^

Pulmonary nocardiosis is the most common presentation as the usual portal of entry for the bacteria is via inhalation. Usually, the onset of symptoms is subacute to chronic as was seen in our case. Common symptoms include productive cough, fever, shortness of breath, chest pain, hemoptysis, night sweats, anorexia, and weight loss. CXR findings can be variable and can present either as a consolidation, cavitary lesion or pleural effusion.^8,9^ The infection can disseminate via hematogenous spread, and bacteremia can be commonly seen in patients with pulmonary nocardiosis as presented in our case. Extrapulmonary presentation is usually an abscess formation, and the most common site is the CNS.^8^ Affected individuals can have one or more brain abscesses and associated pressure like symptoms such as headaches, visual disturbances, and changes in mentation or seizures.^10^ As *Nocardia* is neurotropic, CNS imaging should be considered in immunosuppressed patients with severe disease that was obtained in our case along with a lumbar puncture and showed no CNS involvement. Skin and soft tissue involvement with *Nocardia-*mimicking cellulitis is common in immunocompetent hosts as well. It can result from skin and soft tissue injuries with the contaminated soil. Bacteremia followed by cutaneous infection is less commonly seen. A review of literature from 1980 to 2010 on *Nocardia* infections in renal transplant patients by Yu et al^11^ also reported lung, brain, skin, and subcutaneous tissues as the most frequent organs involved. Kidney allograft, eye, pleura, pericardium, and joints were less commonly involved. *Nocardia* infection occurred more commonly in males after cadaver kidney transplants and developed after a mean duration of 34 months posttransplant. This is in contrast to our case where the *Nocardia* infection developed early on at 4 months posttransplant.

More than 80 species of *Nocardia* have been identified,^12^ of which more than half were described in past 15 years. As per a recent antibiotic susceptibility review of 1299 isolates by Schlaberg et al,^12^ the incidence of *N transvalensis* was only 6%. Yu et al^11^ reported 8 major *Nocardia* species in their review of transplant patients, of which 56% cases were of *Nocardia asteroids*, 18% *Nocardia farcinica*, 9% *Nocardia brasiliensis*, and the rest were under other *Nocardia* species or not classified. Queipo-Zaragozá et al^13^ reported 5 cases of *Nocardia* in a retrospective review of 1239 kidney transplant cases and none were *Nocardia transvalensis. Nocardia transvalensis* infection has been reported in heart transplant recipients,^14^ patients with lung cancer,^15^ patients with cystic fibrosis,^16^ and patients with bronchiectasis and tuberculosis.^7^ McNeil et al reported a renal transplant patient with pulmonary nocardiosis from *N transvalensis* but with an unfortunate outcome.^17^ TMP-SMX has traditionally been considered the gold standard for *Nocardia* therapy, either as monotherapy or as a part of combination therapy particularly for severe infections. The high dose of TMP-SMX, its adverse effects, and now with emerging resistance pattern for some species may limit its use. In the review by Schlaberg et al, antibiotic resistance was most noticeable with *Nocardia pseudobrasilensis* (31%) and *N transvalensis* (19%).^12^ Imipenem/cilastatin is effective against most *Nocardia* species and is often used as the drug of choice in severe cases of disseminated nocardiosis but showed resistance pattern in 94% of isolates of *N transvalensis*^12^ as was clinically evident in our case as well. *N transvalensis* isolates were also found to be highly resistant to ceftriaxone (37%), augmentin (53%), amikacin (72%), ciprofloxacin (16%), minocycline (85%), clarithromycin (96%), and tobramycin (96%).^12^ Drug resistance was very commonly seen in *N transvalensis* isolates (83%), but all the isolates were susceptible to linezolid as shown in our case as well.

Early identification and susceptibility testing of *N transvalensis* could be challenging due to many reasons that include low level of clinical suspicion for *Nocardia*, drug-resistant species, slow rate of growth on cultures, and lack of resources in community hospitals to test for specific *Nocardia* species. However, if clinical suspicion for drug-resistant *Nocardia* species is high, susceptibility testing should be obtained early to reduce morbidity and mortality and to improve clinical outcomes. Overall mortality has been reported to be about 16% to 25%.^12^ The mortality from pulmonary nocardiosis is around 39%, which increased to 64% in disseminated nocardiosis and 100% in the presence of CNS involvement.^18^

## Conclusion

We report a unique case of drug-resistant *N transvalensis* infection with pulmonary involvement within 4 months after cadaveric kidney transplant successfully treated with linezolid and moxifloxacin. Our case illustrates the diagnostic and therapeutic challenges faced in this rare species of *Nocardia* infection. Isolation of *Nocardia* in cultures should not be considered as a contaminant. It could be a life-threatening infection that is difficult to treat requiring protracted course of antibiotics. Guidelines typically recommend 3 to 6 weeks of intravenous antibiotics, followed by 6 to 12 months of oral antibiotics, with even a longer course being used in severe disease and immunocompromised hosts.
